# Type-A response regulators negatively mediate heat stress response by altering redox homeostasis in Arabidopsis

**DOI:** 10.3389/fpls.2022.968139

**Published:** 2022-09-23

**Authors:** Sunita Jindal, Pavel Kerchev, Miroslav Berka, Martin Černý, Halidev Krishna Botta, Ashverya Laxmi, Břetislav Brzobohatý

**Affiliations:** ^1^ Department of Molecular Biology and Radiobiology, Mendel University in Brno, Brno, Czechia; ^2^ National Institute of Plant Genome Research, New Delhi, India

**Keywords:** Arabidopsis response regulators, heat stress, cytokinins, oxidative stress, proteomics, metabolomics, heat-acclimatization

## Abstract

Besides the long-standing role of cytokinins (CKs) as growth regulators, their current positioning at the interface of development and stress responses is coming into recognition. The current evidence suggests the notion that CKs are involved in heat stress response (HSR), however, the role of CK signaling components is still elusive. In this study, we have identified a role of the CK signaling components type-A Arabidopsis response regulators (ARRs) in HSR in Arabidopsis. The mutants of multiple type-A *ARR* genes exhibit improved basal and acquired thermotolerance and, altered response to oxidative stress in our physiological analyses. Through proteomics profiling, we show that the type-A *arr* mutants experience a ‘stress-primed’ state enabling them to respond more efficiently upon exposure to real stress stimuli. A substantial number of proteins that are involved in the heat-acclimatization process such as the proteins related to cellular redox status and heat shock, are already altered in the type-A *arr* mutants without a prior exposure to stress conditions. The metabolomics analyses further reveal that the mutants accumulate higher amounts of α-and γ-tocopherols, which are important antioxidants for protection against oxidative damage. Collectively, our results suggest that the type-A ARRs play an important role in heat stress response by affecting the redox homeostasis in Arabidopsis.

## Introduction

Plants are continuously exposed to environmental temperature fluctuations. Unfavorably high temperature causes heat stress in all organisms and leads to disintegration of membrane lipids, protein misfolding and denaturation, oxidative burst, and increased membrane fluidity. The HS-induced ROS such as $O_{2}^{-}$ and H_2_O_2_ damage organelles and inhibit cellular functions (Szymańska et al., 2017). For example, heat stress negatively influences chloroplast structure, thermal stability of the components of photosystem II and Rubisco activity ultimately resulting in reduced photosynthetic efficiency (Moore et al., 2021). The perception and signaling of heat stress is initiated by high temperature-induced changes in the plasma membrane. The enhanced fluidity of the membrane allows calcium channels to open and an inward flux of Ca^2+^ ions into the cytoplasm, in turn, initiates the heat stress response (HSR) (Abdelrahman et al., 2020).

Heat shock proteins (HSPs) and antioxidant enzymes are the two major classes of functional proteins that are induced during HSR. HSPs work as molecular chaperones and prevent irreversible protein aggregation (Wang et al., 2004). Further, the antioxidant system (AOS) maintains proper cellular levels of ROS through scavenging excessive free radicals. The AOS includes enzymatic components such as superoxide dismutase (SOD), catalase (CAT), ascorbate peroxidase (APX), guaiacol peroxidase (GPX), glutathione reductase (GR) etc., and the non-enzymatic components like ascorbic acid (AsA), reduced glutathione (GSH), α-and γ-tocopherols, carotenoids, flavonoids, and proline (Das and Roychoudhury, 2014) which cumulatively maintain the redox homeostasis.

During heat stress and drought, the phytohormone repertoire is changed markedly and interplays with ROS machinery in mediating stress responses (Xia et al., 2015; Devireddy et al., 2020). In recent years, cytokinins (CKs) have emerged as important phytohormones in mediating heat and water-deficit stress responses alongside their crucial role in growth and developmental plans (Černý et al., 2014; Nguyen et al., 2016; Bryksová et al., 2020; Prerostova et al., 2020). Changes in the endogenous CK levels during several abiotic stresses such as heat and drought prove to be straightforward evidence on the connection of CKs and stress responses (Hare et al., 1997; Todaka et al., 2017). A general trend is that the CK levels rise transiently in response to stress stimuli and decline over time if the stress condition is mild or moderate. In case of increased stress, the CK level remains high or does not return to the baseline level (Zwack and Rashotte, 2015). In addition to the changes in the CK levels, transcriptomic and proteomic studies also reveal a large overlap in the heat stress and CK response profiles in Arabidopsis (Černý et al., 2011; Černý et al., 2014). Further, external application of BAP as well as enhancement of endogenous CK levels by IPT overexpression improve antioxidant capacity in different plant species through activating antioxidant enzymes like CAT and APX (Xu et al., 2016; Hönig et al., 2018) which is crucial for overcoming HS-induced oxidative damage.

Although there is evidence on the involvement of CKs in HSR, the role of CK signaling is currently not understood. The CK signaling module consists of the receptor histidine kinases (AHKs), His-containing phosphotransfer proteins (AHPs) and the type-A and -B response regulators (ARRs). The central components of CK signaling, the type-B ARRs are the transcription factors which are competitively inhibited by the type-A ARRs in a negative feedback loop manner. The type-A ARRs compete with the type-B ARRs for phosphorylation, however, they lack the DNA binding domain for transcriptional activation hence working as the repressors of CK signaling (To et al., 2004). Mutation in the type-A *ARRs*, alleviates the competition for phosphorylation for the type-B ARRs and thus, the repression of the transcription factor type-B ARRs is lifted.

Such negative feedback loops are important for maintaining a delicate balance between the growth and stress response mechanisms and are particularly important under fluctuating environmental conditions (Jamsheer K et al., 2022). Being important growth regulators, it is obvious that the CK signaling is under such regulatory control. Knocking out the negative feedback loop in form of the mutations in type-A *ARRs* thus makes it reasonable to ask how CK signaling in the absence of functional type-A ARRs may influence plants’ capacity to face environmental perturbation such as heat stress. In this study, using detailed physiological analysis we show the role of the type-A ARRs in HSR. In our proteomics studies, we identified that the multiple gene mutations in the type-A *ARRs* lead to an altered proteomic profile which account for the improved survival during the periods of heat stress. Through biochemical assays, together with metabolomic analyses, we also show that the type-A *arr* mutants have modified antioxidant capacity which could contribute to their enhanced ability to survive environmental situations causing oxidative stress.

## Results

### Cytokinin signaling components are differentially regulated by heat stress

**Figure 1:**
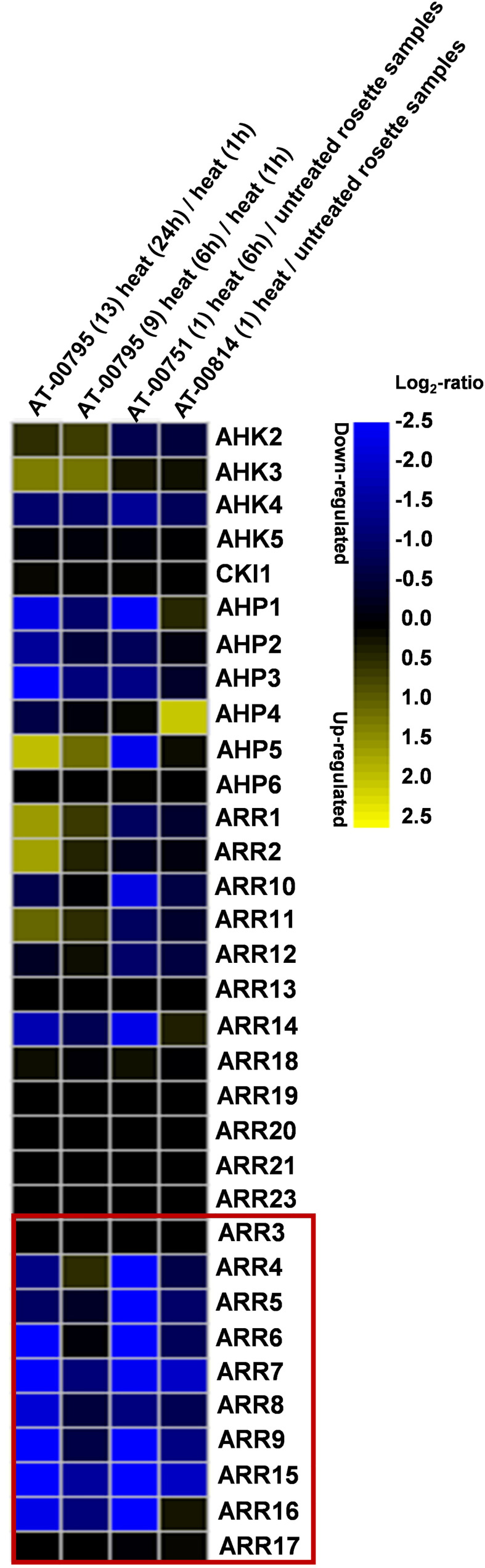
Cytokinin signaling genes are differentially regulated by heat stress. Analysis of the relative expression of *Arabidopsis thaliana* CK signaling genes using the publicly available mRNA-Seq dataset in which Arabidopsis plants were treated with heat stress conditions. The type-A *ARRs* are highlighted in the box. Heat map created using Genevestigator (https://genevestigator.com/).

Since there are several studies indicating the functional link between CKs and HSR, we analyzed the transcriptional regulation of 33 CK signaling component genes under heat stress using publicly available mRNA-Seq dataset “AT_mRNASeq_ARABI_GL-1” from Genevestigator (Hruz et al., 2008). We selected the datasets AT-00814 where heat stress was applied at 35°C for 4 hours to 4-weeks-old Arabidopsis plants, AT-00751 in which 37°C was applied for 6 hours to 3-weeks-old Arabidopsis, and AT-00795 in which 42°C was applied up to 24 h to 3-weeks-old Arabidopsis plants. Out of the 33 analyzed genes, several of them were found to be differentially expressed under heat stress. The highest and most conserved effect was observed on the type-A *ARR* genes. Among these, the expression of 8 out of 10 genes was found to be strongly repressed under heat stress and exhibited a consensus among the different HS treatments (**Figure 1**). In one of the treatments, *AHP4* was upregulated whereas, *AHP1, 2, 5* and *6* were unchanged or slightly changed. In Our analysis, the highest and most conserved response of the type-A *ARR* genes to heat stress indicated their possible involvement in the HSR and therefore, we focused on the type-A *ARR* genes for our subsequent studies.

### Loss of functional type-A *ARRs* improves basal and acquired thermotolerance

**Figure 2:**
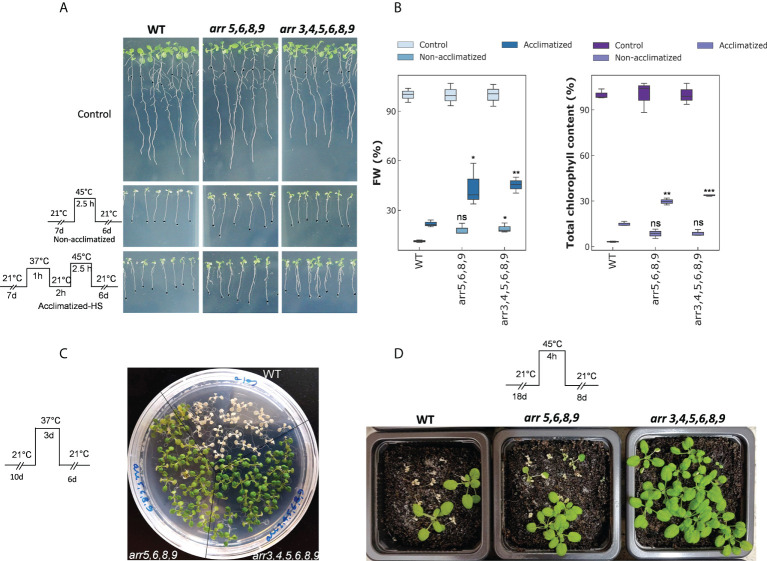
The effect of heat stress on the type-A *arr* mutants. **(A)** Phenotypic response of the 7 DAS (days-after-stratification) seedings subjected to heat stress conditions. Images are captured after 6 DOR (days-of-recovery). **(B)** Plots representing FW per seedling (left) and total chlorophyll content (right). FW and chlorophyll content are normalized with the control samples of each genotype to show the relative differences. The statistical significance is shown between the WT and *arr* mutants within the same treatment group. Error bars represent ± SE (two-way ANOVA, *P-value ≤ 0.05, Bonferroni *post-hoc* test, n=3, 13-15 plants were taken for each biological replicate). Asterisks represent the significant differences between the same treatment group of mutants in comparison to WT. **(C)** Effect of extended and moderate heat stress on 10 DAS seedlings. Images are captured after 6 DOR. **(D)** Effect of severe heat stress on 18 DAS plants. Images are captured after 8 DOR. Other photosynthetic pigments are shown in **Supplementary Figure S1**. ns, non-significant.

Down-regulation of the type-A *ARRs* upon an encounter to heat stress must have adaptive significance for the plants. Therefore, we tested that how type-A *arr* mutants respond to above-optimal high temperatures. Because of the functional redundancy among the type-A ARR family members, most of the single gene mutants are indistinguishable from the wild-type and the double and higher order mutants show increasing sensitivity to CKs (To et al., 2004). Therefore, we selected the higher order Arabidopsis mutants *arr3,4,5,6,8,9* and *arr5,6,8,9* lacking multiple type-A *ARR* genes for our studies. According to To et al., 2004, and in our observation, the quadruple mutant *arr5,6,8,9* was phenotypically indistinguishable from the wild-type under long-day conditions. The *arr3,4,5,6,8,9* mutant is smaller in the rosette area and root length, but we did not observe any differences in the growth progression relative to the wild-type. For basal and acquired thermotolerance assays, the seedlings were treated at a lethal temperature (45°C for 2.5 h) with and without heat acclimatization (37°C for 1 h followed by 2 h of recovery at 21°C) in an incubator. We observed that both the type-A *arr* mutants *arr5,6,8,9* and *arr3,4,5,6,8,9*, showed significantly enhanced thermotolerance in both the conditions (**Figure 2A**). The biomass accumulation after a recovery period of 6 days in the mutants was higher than the wild-type in both conditions, with more prominent effect on the heat-acclimatized plants (**Figure 2B**). Consistently, there was higher total chlorophyll accumulation in the *arr* mutants when normalized with the control growth (**Figure 2B**). Moreover, in the *arr* mutants, chlorophyll *a*/*b* ratio remained closer to control values (**Figure S1**). Also, carotenoid content (normalized with the control growth) stayed higher in the *arr* mutants (**Figure S1**). No statistically significant difference in the total chlorophyll/carotenoid ratio was observed between the wild-type and the *arr* mutants though a trend to decrease in the ratio was apparent in the *arr* mutants (**Figure S1**) as they accumulate carotenoids to higher levels. The heat stress response was further tested in different growth stages and experimental setups. Cultivation at sub-lethal temperature extended to 3 days was found to be lethal for the wild-type while *arr5,6,8,9* and *arr3,4,5,6,8,9* mutants recovered. However, a partial chlorosis was observed in the *arr5,6,8,9* mutant (**Figure 2C**). Various degrees of lethality were observed in all genotypes when soil-grown plants were exposed to a lethal temperature for 4 h. The highest survival rate was observed in *arr3,4,5,6,8,9* mutant followed by *arr5,6,8,9* mutant and the least in the wild-type (**Figure 2D**). Thus, the type-A *arr* mutants displayed significantly higher thermotolerance in all the tested experimental conditions. Collectively, these results identified that the type-A ARRs negatively mediate HSR in Arabidopsis and the lack of functional type-A ARRs leads to improved theromotolerance.

### Differential accumulation of the proteins in the *arr* mutants

**Figure 3:**
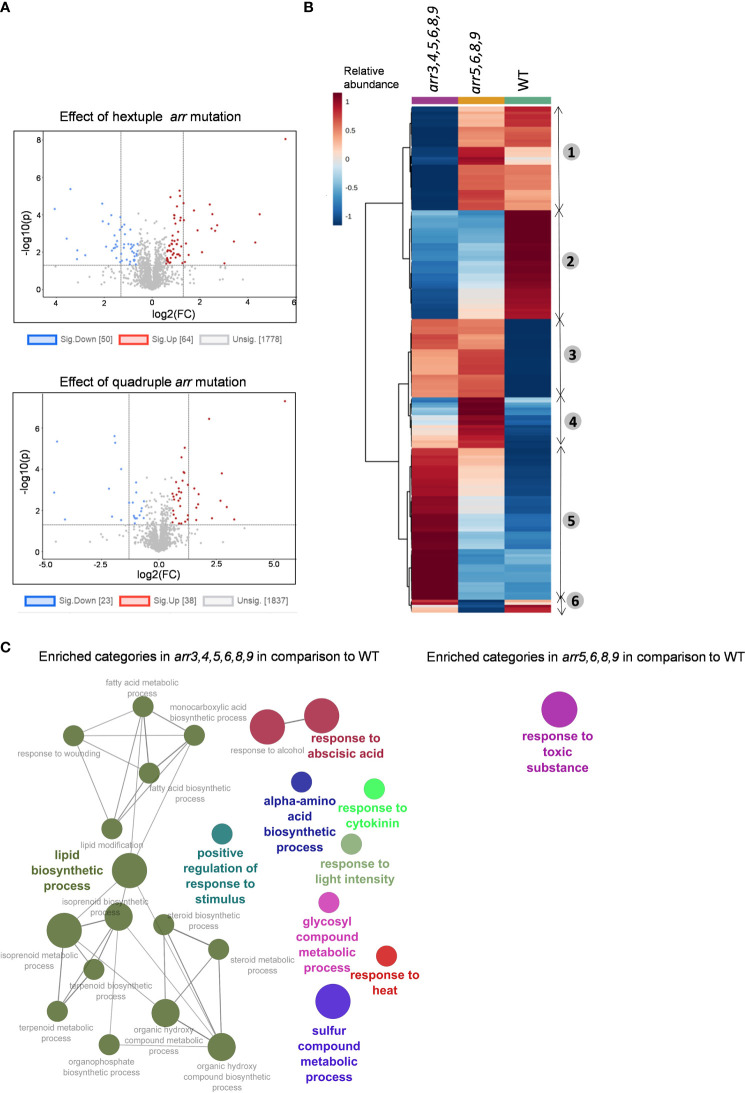
Comparison of the proteome profiles of the *arr* mutants under control conditions. **(A)** Volcano plot visualizations of the protein levels in *arr3,4,5,6,8,9* versus WT (upper) and *arr5,6,8,9* versus WT (lower). FC threshold is set to 1.5 and the P-value threshold is 0.05. **(B)** Hierarchical cluster analysis of the top 200 proteins according to statistical significance. The proteome profiles indicate the additive effect of the loss of type-A *ARR* genes. The quadruple mutant *arr5,6,8,9* shows an intermediate profile with respect to WT and hextuple mutant *arr3,4,5,6,8,9*. The heat map visualization and hierarchical clustering is carried out using MetaboAnalyst 5.0 and the numbers next to the heat map indicate six different clusters based on similarity in the accumulation pattern. **(C)** GO term functional grouping of the DAPs in the *arr* mutants as compared to WT. The network visualization is done in Cytoscape v3.9.0 plugin Clugo. The leading group name is based on the highest significance and only the pathways with a P-value ≤ 0.05 are shown. The nodes represent the GO terms and the size of the node represents term enrichment significance. At least 3 biological replicates are analyzed. **(A, C)**, The list of DAPs and proteins in enriched GO terms are given in **Supplementary Table S1** and **S2**.

To investigate the effect of mutations in the type-A *ARR*s on the overall proteomic landscape and subsequently its effect on HSR, we conducted shotgun proteomics analyses from the wild-type and the type-A *arr* mutants *arr5,6,8,9* and *arr3,4,5,6,8,9*. Since the heat treatment at 45°C had severe negative effect on young seedling survival post-recovery, and the differences in the theromotolerance of the *arr* mutants compared to the wild-type were more prominent in heat-acclimatized groups (**Figure 2A**), we got interested to know what happens differently in the *arr* mutants after a short heat-acclimatization phase before they encounter a severe heat stress. For this, we treated 7 DAS (days-after-stratification) Arabidopsis seedlings at 37°C for 1 h and harvested them after 2 h of recovery at 21°C. For the untreated groups, 7 DAS seedlings were harvested at the same time point as the heat-treated group. From all the measured spectra, peptides matching 4657 *Arabidopsis thaliana* proteins were identified. Out of these, 3310 proteins with at least two unique peptides were selected for further analyses. First, we compared the proteome profiles of *arr5,6,8,9* and *arr3,4,5,6,8,9* mutants with respect to the wild-type under unstressed conditions to unravel the type-A ARR-regulated proteome and to see the difference on the impact of additional *arr* mutations. In the hextuple mutant, we identified a total number of 114 differentially accumulated proteins (DAPs) of which, 64 were up-regulated and 50 were down-regulated (**Figure 3A**). In the quadruple mutant, on the other hand, only 61 proteins were differentially accumulated where the number of up- and down-regulated DAPs was 38 and 23, respectively (**Figure 3A**), suggesting an additive effect of the *arr* mutations on the proteome. A complete list of the DAPs is given in **Supplementary Table S1** and **S2**. To compare the accumulation patters of proteins in both the mutants, we constructed a heat map. Hierarchical clustering of the top 200 proteins according to statistical significance highlighted clustering of these proteins into 6 groups (**Figure 3B**). In the clusters 1 and 5, the accumulation patters of the proteins were similar in the *arr5,6,8,9* and wild-type compared to the *arr3,4,5,6,8,9.* In clusters 2 and 3 the accumulation patters were similar in both the mutants compared to the wild-type. Clusters 4 and 6 had a smaller number of proteins for which accumulation levels were closer in the hextuple mutant and wild-type. This observation suggested that the proteome profile of the quadruple mutant was intermediate to that of the wild-type and *arr3,4,5,6,8,9* in terms of the number of DAPs as well as the level of accumulation as can be seen in the heat map. Due to the partially redundant functions of the gene family, the effect of the multiple gene mutations could be largely additive as appears from this result, however, the functional specificity of the particular gene family members cannot be ruled out. The additive effect of the lack of functional type-A *ARR* genes reflects in the physiological response of these mutants under HS conditions as the hextuple mutant exhibited higher thermotolerance than the quadruple mutant in our visual assessment (**Figure 2**).

Further, we carried out the gene ontology (GO) enrichment analysis of DAPs in both the mutants using Cytoscape v3.9.0 plugin Clugo (Bindea et al., 2009) (**Figure 3C**). In the hextuple mutant, the highest number of proteins (13 each) corresponded to the categories, ‘response to abscisic acid’ and ‘lipid biosynthetic process’, followed by ‘sulfur compound metabolic process’ (10). The other enriched GO terms were ‘response to cytokinins’, ‘response to heat’, ‘alpha-amino acid biosynthetic process’, ‘response to light intensity’, ‘response to stimulus’ and ‘glycosyl compound metabolic process’. In quadruple mutant *arr5,68,9*, the only enriched GO term was ‘response to toxic substance’ (**Figure 3C**). The list of proteins in the enriched GO terms is given in **Supplementary Table S1 and S2**. The enrichment of the proteins involved in the response to cytokinins indicated an enhanced CK signaling in the *arr3,4,5,6,8,9* mutant. We observed that several proteins related to heat and/or oxidative stress response such as HOP3 (HSP70-HSP90 organizing protein 3), HSP17.4B/HSP17.6A, LIPOXYGENASE1 (LOX1), Peptidemethionine Sulfoxide Reductase 1 (PMSR1), Glutathione peroxides 2 (GPX2), Glutathione S-transferase theta 1 (GSTT1), Glutathione S-transferase U1 (GSTU1), ANNEXIN4 (ANN4), peroxidase 34 (PER34), a putative peroxidase AT4G26010.2, glutaredoxin and thioredoxin family proteins, and a respiratory chain NADH dehydrogenase were altered in one or both the mutants. A list of redox-related proteins that are differentially accumulated in the *arr* mutants in comparison to the wild-type are given in **Supplementary Table S5**. Many of the genes encoding these proteins are known oxidative stress marker genes. The higher accumulation of these proteins in the *arr* mutants indicated that the *arr* mutants experience a ‘stress-primed’ state which could be responsible for their increased thermotolerance upon a real encounter with elevated temperatures.

### Acclimatory heat-regulated proteins are altered in the *arr* mutants prior to heat exposure

**Figure 4:**
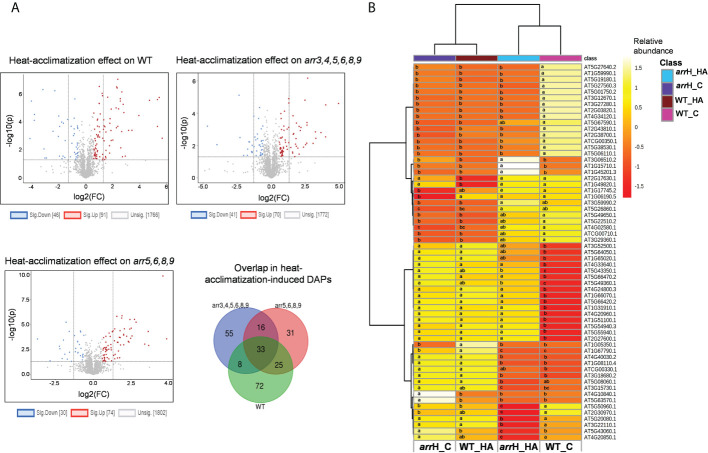
Proteomics changes triggered by heat-acclimatization in WT and *arr* mutants. **(A)** Comparison of the heat-acclimatization-induced proteomic changes in the WT and *arr* mutants. The 7 DAS seedlings were treated at 37°C for 1 h and the samples were harvested after a 2 h recovery phase at 21°C. FC threshold is set to 1.5 and the P-value threshold is 0.05. and **(B)** Heat map view of DAPs upon heat-acclimatization/mild heat treatment. Only the proteins that show statistical interaction in two-way ANOVA (genotype x treatment) at P-value ≤ 0.05 are shown in the heat map and the letters on the heat map indicate statistical significance in Turkey’s test. The protein identities are listed on the right. The heat map scale indicates relative abundance. Treatments were performed in at least 3 biological replicates. The heat map visualization and hierarchical clustering are carried out using MetaboAnalyst 5.0. arrH_C: *arr3,4,5,6,8,9*_Control; arrH_HA: *arr3,4,5,6,8,9*_Heat-acclimatized; WT_C: WT_Control; WT_HA: WT_Heat-acclimatized. The list of DAPs upon heat-acclimatization in WT is given in **Supplementary Table S3**. The list of the proteins showing interaction effect in two-way ANOVA is given in **Supplementary Table S4**. The heat map view of the comparison of WT and *arr5,6,8,9* proteomes upon heat-acclimatization is given in **Supplementary Figure S3**.

To further explore the basis of enhanced thermotolerance of the *arr* mutants, we analyzed how a heat-acclimatization phase triggers proteomic reprogramming differently in the *arr* mutants in comparison to the wild-type. We observed that the overall proteomic changes in the wild-type were higher than both the *arr* mutants as a total number of 137 proteins were significantly differentially accumulated in wild-type versus 111 and 104 in *arr3,4,5,6,8,9* and *arr5,6,8,9*, respectively (**Figure 4A**), indicating that heat treatment at 37°C induces more robust changes in the proteome in the wild-type than the *arr* mutants. The GO enrichment analysis of the heat-regulated DAPs in the wild-type seedlings retrieved the enriched functional categories such as ‘heat acclimation’, ‘response to heat’, ‘response to hydrogen peroxide’, ‘sulfur compound metabolic process’, ‘organic acid metabolic process’ and ‘organophosphate biosynthetic process’ (**Figure S2A**, **Supplementary Table S3**). The enrichment of the proteins related to heat and oxidative stress validated the heat treatment. Interestingly, some of the functional categories viz. ‘response to heat’, ‘response to light intensity’ and ‘sulfur compound metabolic process’ were also enriched in the *arr3,4,5,6,8,9* mutant as compared to the wild-type under control conditions indicating an overlap between the heat-induced proteome and the type-A ARRs-regulated proteome. Further, multiple factors/covariates data analysis was carried out to compare the proteomic response of wild-type and *arr3,4,5,6,8,9* upon heat-acclimatization. In two-way ANOVA analysis, a total number of 1687 proteins were found to be differentially accumulated, of which, 61 showed interactions (genotype x treatment). Intriguingly, the heat map visualization of these 61 proteins revealed that a high proportion of these proteins were already altered in the *arr3,4,5,6,8,9* mutant prior to heat-acclimatization and remained more stable after the short acclimatization phase (**Figure 4B**). Therefore, the proteome profile of the unstressed *arr3,4,5,6,8,9* significantly resembled the heat-acclimatized wild-type seedlings. The GO enrichment analysis of these 61 proteins showed the enrichment of biological function categories ‘nucleobase-containing small molecule metabolic process’, ‘alpha-amino acid biosynthetic process’ and ‘response to metal ion’ etc. (**Figure S2B**, **Supplementary Table S4**). A closer protein-by-protein look revealed that two cell-division and meristem development-related proteins Prohibitin4 (PHB4) and ZUOTIN-RELATED FACTOR1B (ZRF1B; AT5G06110.1) were repressed in the wild-type after heat-acclimatization phase. In the mutant, both these proteins were already maintained at a lower level and did not decline after treatment (**Figure S2C**). Similar accumulation pattern was observed for the CBS DOMAIN CONTAINING PROTEIN 2 (CBSX2). CBSX2 regulates thioredoxins which participate in the cellular redox system (Yoo et al., 2011). In contrast, several proteins directly or indirectly implicated in the oxidative and/or heat stress response such as the PYRIMIDINE DEAMINASE (PYRD), GPX2, GSTT1, PER57 and HOP3 were induced in the wild-type plants following an exposure to 37°C and short recovery period, whereas, in the *arr3,4,5,6,8,9* mutant, the levels of these proteins under control condition were already high and did not change as much as wild-type upon the heat treatment (Fig S2C). Proteins such as DJ1A (involved in oxidative stress), GLYOXALASE12 (ATGLYI2) and Arabidopsis Aldehyde Oxidase2 (AAO2), on the other hand, exhibited opposite induction pattern in the wild-type and the *arr3,4,5,6,8,9* mutant. ATGLYI2 is the first enzyme of the glyoxalase pathway through which methylglyoxal (MG), a toxic byproduct of glycolysis is detoxified. MG levels rise under abiotic stress, therefore the higher abundance of ATGLYI2 in the *arr3,4,5,6,8,9* mutant could possibly improve their capacity to detoxify stress-induced MG. We extended this analysis to the quadruple mutant *arr5,6,8,9* and found out that similar to the hextuple mutant *arr3,4,5,6,8,9*, some heat-regulated proteins are already altered in this mutant, however, the number of such proteins was relatively lower compared to the hextuple mutant which is in line with our other observations about the intermediate position of the *arr5,6,8,9* in the HSR and proteomic changes (**Figure S3**). Collectively, these results identified that the lack of functional type-A ARRs induces similar proteomic changes as that of a heat-acclimatization phase in wild-type for several proteins involved in heat and oxidative stress.

### Metabolic responses due to elevated temperatures

**Figure 5:**
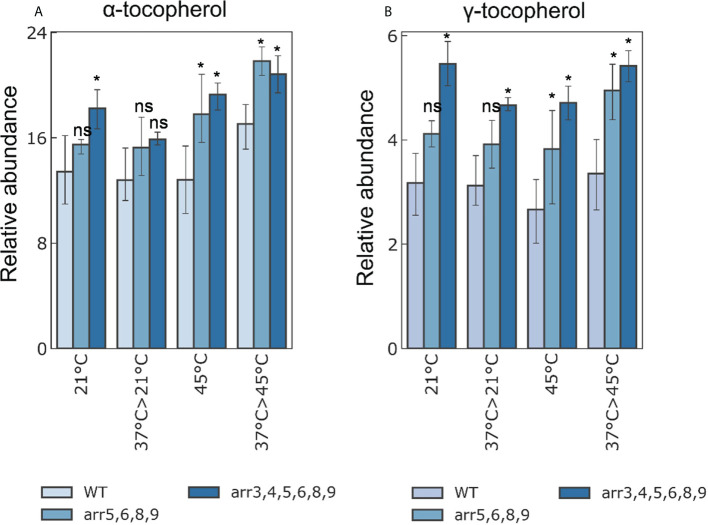
Relative accumulation of **(A)** α- and **(B)** γ-tocopherols under different temperature conditions. The 7 DAS seedlings were treated at different heat-stress conditions and samples were harvested for metabolomics analyses (21°C: Control; 37°C>21°C: 37°C for 1 h followed by 2 h recovery at 21°C; 45°C: 45°C for 2.5 h; 37°C>45°C: 37°C for 1 h followed by 2 h recovery at 21°C and 45°C for 2.5 h). The bars shown are the average of four biological replicates. Error bars represent ± SE (two-way ANOVA, *P-value ≤ 0.05, Bonferroni *post-hoc* test, n=3, 13-15 plants were taken for each biological replicate, ns not significant). The statistical differences shown in the graphs are the comparison of the *arr* mutants with respect to WT in the respective treatment group. A detailed overview of all the analyzed metabolites is given in **Supplementary Figure S4**. The tocopherol biosynthetic pathway and the relative expression levels of the pathway genes are shown in **Supplementary Figure S5**.

Determination of the changes in metabolite dynamics provides a more conclusive picture of the physiological reprogramming in a system-wide manner. Therefore, we determined the changes in non-polar and polar metabolites accumulation in the Arabidopsis seedlings. We used the same samples which were used for proteomics analyses in which 7 DAS seedlings were subjected to a heat-acclimatization phase and mapped 60 polar and 36 non-polar metabolites. Our analysis revealed that a mild heat treatment/heat-acclimatization does not reflect at the level of metabolites in any genotype probably either because a brief exposure of 1 h at 37°C was too subtle to bring about a metabolomic response or due to the reason that we monitored only the rapidly induced metabolic changes as we harvested the samples after 2 h of recovery at 21°C. Nonetheless, we extended our analysis at higher temperature stress at 45°C with and without the acclimatization phase. Among the polar metabolites, we found out that, several sugars, sugar alcohols, amino acids and amines were promptly accumulated in response to severe heat stress irrespective of the acclimatization phase and genotype, however, this response was similar in the wild-type and the mutants (**Figure S4A, B**). As a biochemical response to HS, sugars are known to accumulate due to their osmoprotectant function (Melandri et al., 2021), and this observation validates our HS application. Among the non-polar metabolites that we mapped, an interesting observation was the higher accumulation of protective compounds α- and γ-tocopherols in both *arr5,6,8,9* and *arr3,4,5,6,8,9* mutants (**Figure 5**). To get a closer insight into the preferential accumulation of α- and γ-tocopherols in the *arr* mutants, we determined transcript levels of genes involved in tocopherol biosynthesis (**Figure S5**). The higher levels of α- and γ-tocopherols in the *arr* mutants correlated with the up-regulation of *VTE2, VTE3* and *VTE1* in the *arr3,4,5,6,8,9* mutant. *VTE2*, *VTE3* and *VTE1* encode the enzymes HGA phytyltransferase, a methyltransferase and a tocopherol cyclase, respectively, and due to their higher expression, the metabolic flux is probably directed more preferentially towards the biosynthesis of α- and γ-tocopherols. Similar to the proteomics data, *arr5,6,8,9* accumulated intermediate levels of α- and γ-tocopherols as compared to the wild-type and *arr3,4,5,6,8,9*. This trend was observed in all the temperature conditions and both the mutants accumulated higher amounts of α- and γ-tocopherols. α-tocopherol is known for its antioxidant activity and prevents lipid peroxidation by scavenging lipid peroxyl radicals in thylakoid membranes thus protecting the photosynthetic apparatus, while γ-tocopherol is predominantly found in the seeds and can be functionally replaced for α-tocopherols (Munné-Bosch, 2005; Munné-Bosch, 2019). A higher accumulation of tocopherols in the *arr* mutants is a strong indication of higher antioxidant capacity and supports our proteomics data. Taken together, our analyses revealed that heat stress does not differentially induce the metabolomic changes in the *arr* mutants, however, we found out that the mutants accumulate higher amounts of protective antioxidant compound.

### The type-A *arr* mutants show altered response to oxidative stress conditions

**Figure 6:**
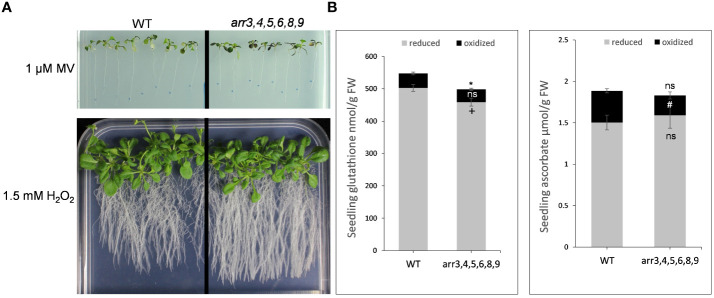
Oxidative stress response and cellular redox alterations in the absence of functional type-A ARRs. **(A)** Phenotypic response of Arabidopsis seedlings under oxidative stress conditions. 7 DAS seedlings grown on 0.5 X MS medium were transferred to the medium containing methyl viologen (MV) or H_2_O_2_. The images are captured after 2 weeks (for MV treatment) and 3 weeks (for H_2_O_2_ treatment) of transfer to the media containing MV or H_2_O_2_. The treatments were carried out in 4 biological replicates. **(B)** Glutathione and ascorbate content in WT and *arr3,4,5,6,8,9* mutant. Grey bars indicate the reduced forms of glutathione (GSH) or ascorbate (ASC) content, and black bars indicate the oxidized forms glutathione disulfide (GSSG) or dehydroascorbate (DHA). Average from four biological replicates are shown and the error bars indicate ± SE. Asterisk indicates a significant difference in total glutathione and ascorbate, + indicates a significant difference in reduced forms, and # indicates a significant difference in the oxidized forms between the wild-type and *arr* mutant P-value ≤ 0.05. ns, non-significant.

Our proteomics and metabolomics results strongly suggested links between the type-A ARRs and the redox-regulation machinery due to which the type-A *arr* mutants can better cope the heat stress-induced oxidative burst. We, therefore, tested the effect of direct oxidative stress on the plants by transferring the seedlings in the medium supplemented with various concentrations of hydrogen peroxide (1.0, 1.5, 4.5 mM H_2_O_2_) and methyl viologen (0.1, 0.5, 1.0 µM MV), an herbicide that induces ROS formation. We sealed the plates with parafilm and cling film to allow the accumulation of ROS. As the behavior of both the mutants were similar, we conducted this experiment on the higher order *arr3,4,5,6,8,9* mutant. As we anticipated, the *arr3,4,5,6,8,9* mutant exhibited an altered response to oxidative stress conditions after prolonged growth in the same sealed plates. We did not observe any difference in the physiological response of the mutant seedlings at the lower concentrations of MV. The highest tested concentration 1 µM MV was extremely inhibitory for all the seedlings, however, in our visual assessment, we observed that the *arr* mutant accumulated higher anthocyanins in the leaves and showed reduced chlorosis as compared to the wild-type. Further, under 1.5 µM H_2_O_2_, mutant plants exhibited a more robust root system and stress-induced flowering was delayed as compared to the wild-type (**Figures 6A**, **S6**).

In our GO enrichment analyses of the differentially accumulated proteins, we found an enrichment of the proteins functioning in ‘sulfur compound metabolic processes’ and ‘oxidative stress’ in the *arr* mutant. For example, GPX2, GSTT1, GSTU1, glutaredoxin and thioredoxin family proteins were differentially accumulated in the absence of functional type-A ARRs. Literature evidence also suggests that CKs can mediate the ascorbate-glutathione (ASC-GSH) pathway which are in the core of the redox hub (Foyer and Noctor, 2011; Porcher et al., 2021). This promoted us to ask if the type-A ARRs are also involved in the ascorbic acid-glutathione (AsA-GSH) pathway. We estimated the glutathione (GSH/GSSG) and ascorbate (ASC/DHA) contents in 10 DAS wild-type and *arr3,4,5,6,8,9* seedlings. We found out that the level of total and reduced glutathione was lower in the *arr* mutant as compared to the wild-type (**Figure 6B**). While there was no significant difference in the level of total ascorbate, the oxidized form was significantly lower in the *arr* mutant compared to the wild-type (**Figure 6B**). These results, together with the metabolomic studies indicated that the type-A ARRs modulate the dynamics of the AsA-GSH-tocopherol triad.

## Discussion

In this study, we identified the role of type-A ARRs in plant HSR. Several studies have previously indicated the involvement of CKs in abiotic stress responses. CK levels and stress conditions are shown to mutually affect each other. For example, high or low temperature stresses have been reported to modulate the endogenous CK levels (Hare et al., 1997; Todorova et al., 2005; Todaka et al., 2017; Prerostova et al., 2020) and, at the same time, exogenous or endogenous increase in the CK levels can improve antioxidant capacity in plant (Xu et al., 2016; Hönig et al., 2018; Berka et al., 2020). It is also reported that the molecular responses to heat and CK overlap (Černý et al., 2014). However, how various CK signaling components participate in the HSR is currently not known. Here, we show that the type-A ARRs negatively regulate HSR by altering the redox state of the plant.

While individual ARRs might have evolved to perform specialized functions, they are known to function in a partly redundant manner (To et al., 2004; Salomé et al., 2006). We observed that the physiological responses of the quadruple mutant were also subtle in comparison to the hextuple mutant. Notably, long-term exposure to sub-lethal temperature proved to be lethal for the wild-type, partial chlorosis was apparent in *arr5,6,8,9* mutant and *arr3,4,5,6,8,9* mutant recovered without any significant damage. Chlorosis is a part of leaf senescence. Heat-induced leaf senescence is stimulated by heat-induced levels of abscisic acid (ABA) and inhibited by type-B ARRs (Zhang et al., 2017). Activity of type-B ARRs is inhibited by a negative feedback regulation *via* type-A ARRs which is reflected in increasing sensitivity to CK in higher order type-A *arr* mutants (To et al., 2004). Thus, the absence of any significant heat-induced leaf senescence in *arr3,4,5,6,8,9* mutant might result from the highest activity of type-B ARRs fully counteracting ABA-induced senescence. Similarly, in our proteomics analyses, the number of DAPs and the extent of accumulation of certain DAPs was intermediate in the *arr5,6,8,9* mutant as compared to the wild-type and the *arr3,4,5,6,8,9* mutant. Such kind of additive effect is largely common for the members of a gene family. For example, the triple mutations in the type-B *ARR* genes cause a more prominent effect on drought tolerance as compared to double gene mutations (Nguyen et al., 2016).

Our proteomics analyses revealed that several proteins involved in the growth, metabolism, and stress responses were altered in the mutants lacking the type-A *ARR* genes and the hextuple *arr* mutant sported a strikingly similar proteomic landscape that the wild-type plants acquire upon heat-acclimatization. This indicates the preparedness of these mutants to face the high temperature challenge. For example, higher accumulation of a riboflavin biosynthetic enzyme PYRD in the *arr3,4,5,6,8,9* mutant could positively affect the riboflavin content. Riboflavin acts as a stress-priming compound in plants and possesses antioxidant properties, and its moderately increased levels have been associated with the improved AOS and drought tolerance in Arabidopsis (Deng et al., 2014). Further, peroxidases/glutathione peroxidases (GPXs) and glutathione S-transferases (GSTs) are well-recognized for their positive role in abiotic stress tolerance due to their ROS-scavenging activity and thus they play important roles in determining cellular redox environment (Roxas et al., 1997; Liu et al., 2013; Zhang et al., 2019). Mostly higher accumulation of ferredoxin/thioredoxin family protein, peroxidase family protein, GPX2, GSTT1, GSTU1, NADH dehydrogenase-related protein and superoxide dismutase protein in one or both the *arr* mutants indicates and altered cellular redox environments in these mutants. Similarly, PMRSs that are present in all the sequenced organisms, repair oxidatively damaged proteins (Gustavsson et al., 2002; dos Santos et al., 2005). It is important to note that PMSR is referred as one of the minimal set of proteins sufficient for cell life (Mushegian and Koonin, 1996), highlighting its important position. The HOP family proteins work as cytosolic co-chaperones and play a major role in long-term acquired thermotolerance. The most heat-regulated of these is the HOP3 (Fernández-Bautista et al., 2018). The proteins discussed here are accumulated in higher amounts in one or both the type-A *arr* mutants even under control conditions. In addition, two cell-division and meristem development-related proteins PHB4 and ZRF1B are repressed upon heat-acclimatization in the wild-type plants. The already low level of these two proteins in the *arr3,4,5,6,8,9* mutant could be a contributing factor to the higher resistance of the mutant under heat stress as moderation of cell-division is an adaptive feature in fluctuating environments (Zhang et al., 2020). Taken together, our results indicated that the cellular environment of the *arr* mutants is more protective against the heat stress-induced oxidative damage, and it could be the major contributing factor for its improved theromotolerance in addition to the cell-cycle moderation. It will be interesting to further explore the molecular connections between the type-A ARRs and the proteins involved in heat-acclimatization process.

NADH dehydrogenases are important components of the electron transport chain in all the organisms. NADH dehydrogenase as well as NADH-dependent reductases are involved in the maintenance of the redox state (Schertl and Braun, 2014; Hyun and Lee, 2015). In our proteomics analyses, we found that 2 NADH dehydrogenases (FRO1 and AT4G02580.1) and a NADH-dependent reductase, CBR2 show differential accumulation upon heat-acclimatization in the *arr* mutant as compared to the wild-type. FRO1, also known as NDUFS4 is an NADH-ubiquinone oxidoreductase-like protein and mutation in the gene encoding this protein leads to constitutively reduced phosphorylation efficiency. The cellular metabolism adapts to this situation and provides moderate stress tolerance to plants (Meyer et al., 2009). In our analysis, heat-acclimatization leads to reduction in the level of FRO1 in the wild-type, whereas it is maintained at lower levels in the *arr* mutant indicating a preset adaptability in the mutant.

Tocopherols, commonly referred as vitamin E, are implicated in several abiotic stresses (Munné-Bosch, 2019). The Arabidopsis *vte1* and *vte4* mutants which are deficient in α-and γ-tocopherols show increased sensitivity to salt stress and accumulate higher levels of H_2_O_2_ and malondialdehyde (MDA) (Ellouzi et al., 2013). Increasing the levels of tocopherols through alteration in the biosynthetic pathway has been shown to improve the antioxidant capacity and abiotic stress tolerance in plants (Kanwischer et al., 2005; Abbasi et al., 2007; Yusuf et al., 2010; Ma et al., 2020). The higher accumulation of the tocopherols in the type-A *arr* mutants under unstressed as well as heat stress conditions is certainly an important feature by which the mutants resist or overcome the heat stress-induced damage. The quantitative effect on the *arr* quadruple and hextuple mutants on the accumulation of tocopherols indicate that the type-A ARRs regulate tocopherol biosynthesis in a functionally redundant manner. Our relative gene expression profiling of the tocopherol biosynthesis pathway genes reveals that *VTE2*, *VTE3* and *VTE1* are up-regulated in the *arr3,4,5,6,8,9* mutant and more studies in this direction will be interesting to dissect the type-A ARR control over the tocopherol biosynthesis.

Glutathione and ascorbate are widely known for their positive effect on various abiotic stress conditions and in maintaining redox homeostasis (Foyer and Noctor, 2011; Kerchev and van Breusegem, 2022). Glutathione is a major non-protein sulfur-containing compound. In our proteomics analysis, we found an enrichment of the proteins related to sulfur compound metabolic process in the type-A *arr* mutant. Our biochemical assays revealed that the total glutathione levels were lower in the *arr* mutant. While this observation seems counterintuitive because glutathione has a protective role in oxidative stress, we can assume that the type-A ARRs regulate the ASC-GSH-tocopherol axes in a selective manner. In line with our results, it has been shown previously that the overall antioxidant capacity is reduced in the plants with reduced CK-status due to mutation in CK receptor *AHK* genes and induction of CK degradation (Cortleven et al., 2014). In addition, in our independent study (in-communication), we found out that the activation of CK signaling differentially regulates the sulfur-starvation marker genes and is accompanied by a decrease in sulfate and GSH levels. In contrast, CK-deficient mutants were found to maintain higher GSH content and displayed enhanced tolerance to GSH-depleting agents in root growth assay strongly suggesting a CK-sulfur interplay in regulating plant growth and stress responses. In the CK-deficient line *35S:CKX4*, which have increased CK degradation, accumulated higher amount of glutathione in control conditions and has almost no difference in the level of ascorbate. However, it had overall lower antioxidant capacity because of the different status of other antioxidant compounds (Cortleven et al., 2014). This indicates the negative impact of the CK-status on the glutathione accumulation. The precise functions of the CK signaling components in the antioxidant system and abiotic stress protection are yet to be defined, nonetheless, our study provides a starting point to unravel the molecular basis of the role of CK signaling in high temperature stress responses.

Photosynthetic pigment contents are known to drop in response to heat stress (García-Plazaola et al., 2017; Wang et al., 2018). Therefore, the pigment contents and their ratios can serve as indicators of stress tolerance. In our study, no statistically significant difference in chlorophyll and carotenoid contents was found between the wild-type and *arr* mutants under control conditions. However, the pigments were retained in the *arr* mutants at higher levels following heat stress treatments in accordance with the sensitized CK signaling in these mutants. The increase in pigment retention was further enhanced in acclimatized *arr* mutants. Previously, stimulation of CK biosynthesis was shown to result in increased contents of chlorophyll *a* and *b*, and carotenoids in transgenic tobacco (Cortleven and Valcke, 2012). Higher chlorophyll *a* retention in the *arr* mutants is in line with a finding in rice where CK treatment was shown to delay dark-induced senescence by accumulation of 7-hydroxymethyl chlorophyll, a precursor of chlorophyll *a*, and up-regulation of genes of the chlorophyll cycle which converts chlorophyll *a* to chlorophyll *b*, thereby maintaining chlorophyll *a/b* ratio (Talla et al., 2016). The higher retention of chlorophyll *a*/*b* ratio might indicate lower content of light-harvesting chlorophyll *a*/*b*-binding proteins (LHC) associated with PSII (LHCII) to transfer excitation energy to the PSII core complex (Xu et al., 1995). The decrease in LHCII resulting in reduction of light absorption cross-section of photosystems is an essential protection mechanism which allows plants to survive unfavorable conditions (Lichtenthaler, 1987; Špundová et al., 2003). Further, it is well established that carotenoid functions other than to light harvesting include photoprotection by either protecting photosynthetic systems against ROS or singlet energy dissipation by non-photochemical quenching (Young and Young, 1991; Loggini et al., 1999; Caffarri et al., 2001). Thus, the sensitized CK signaling in the type-A *arr* mutants contributes to both basal and acquired thermotolerance in Arabidopsis, at least partly, *via* suppression of heat-induced leaf senescence through stimulation of chloroplast protection or recovery and by enhancing the pool of low molecular weight antioxidants through increased carotenoid content.

## Materials and methods

### Plant material and growth conditions

The *Arabidopsis thaliana* Columbia-0 (Col-0) ecotype was used as wild-type control for all experiments. The quadruple mutant *arr5,6,8,9* and hextuple mutant *arr3,4,5,6,8,9* both are in the Col-0 background. The *arr5,6,8,9* (N25277) and *arr3,4,5,6,8,9* (N25279) mutant lines were obtained from NASC. The seeds were surface sterilized and stratified in dark for 3 days at 4°C and grown on 0.5X Murashige & Skoog medium (pH: 5.7) supplemented with 1% w/v sucrose and 0.8% w/v agar at 21°C and long day conditions (16 h light, 60 μmol m^-2^ s^-1^ PPFD provided by Philips TL-D fluorescent tubes) in a climate-controlled growth chamber (AR36LX, Percival, http://www.percival-scientific.com/) with 70% relative humidity. This media composition and growth conditions are used in all the experiments unless specified. The experiments on adult stages were performed in 5 cm tall pots filled with soil and perlite (1:1).

### Stress treatments

For thermotolerance assay, the seedlings were initially grown at standard conditions as mentioned above for 7 days unless specified. To assess basal and acquired thermotolerance, we conducted experiments on two separate groups namely non-acclimatized and acclimatized. The seedlings of non-acclimatized group were treated with a lethal temperature 45°C for 2.5 h followed by recovery for 3-7 days at standard growth conditions. The acclimatized group was first exposed to a sub-lethal temperature 37°C for 1 h to initiate an acclimatory response, followed by 2 h of recovery at 21°C then transferred to 45°C for 2.5 h and subsequently allowed to recover along with the non-acclimatized group. The heat treatments were carried out in an incubator (BINDER GmBH, Germany) with the same light quantity as in the control conditions. A control group was grown in parallel at standard growth conditions. To access the effect of extended exposure at sub-lethal temperatures, wild-type and mutant seedlings were grown in the same plates for 10 days after stratification and then kept at 37°C for 3 days and returned to standard growth conditions. HS on the 18 DAS soil-grown plants was applied at 45°C for 4 h.

The oxidative stress treatment was carried out by transferring the 7 DAS seedlings to the media containing methyl viologen (0.1, 0.5 and 1 µM), and H_2_O_2_ (1.0, 1.5 and 4.5 µM). The plates were sealed with parafilm to maintain the oxidative environment and allowed to grow at standard growth conditions. The images were captured using Canon EOS 600D camera.

### Measurement of stress-regulated biomass accumulation and content of photosynthetic pigments

The fresh weight of seedlings was measured on a microbalance. For chlorophyll and carotenoid estimation, the seedlings were placed in 80% (v/v) acetone at 4°C for overnight in the dark. Cell debris was pelleted by centrifugation at 7800 g for 10 min and the amount of chlorophyll *a* (12.21×A663-2.81×A646), chlorophyll *b* (20.13×A646-5.03×A663) and carotenoids [(1000×A470-1.82×Chl*a*-85.02×Chl*b*)/198] was measured at the wavelengths 663 and 646 and 470 nm using a spectrophotometer (Lichtenthaler and Wellburn, 1983; Lichtenthaler and Buschmann, 2001). The total amount of chlorophyll (Chl*a* + Chl*b*) and carotenoids was then quantified and normalized per seedling. For phenotypic analysis, the digital images were captured using Canon EOS 600D camera.

Metabolite extraction and GC-MS analysis

Arabidopsis seedlings were grown and treated as mentioned in the thermotolerance assay. An equal amount of plant tissue (50 mg) was harvested in four biological replicates. Samples were extracted in precooled MTBE : MeOH (3:1, v:v) mixture, spiked with 0.05 μg ml^-1^ of valine C13 and the extracts were vortexed followed by ice-cooled sonication for 15 min. The samples were centrifuged for 10 min at 17,000 g at 4°C and the supernatant was transferred to the new 2 ml microcentrifuge tubes and maintained on ice. An equal volume of H_2_O:MeOH (3:1, v:v) was added and the samples were vortexed and finally centrifuged for 10 min at 17000 g at 4°C. Two aliquots from polar and nonpolar phase (100 μl) were taken to dryness in a speed vacuum concentrator and the dried residues were redissolved and derivatized for 90 min in 20 μL of 40 mg mL^-1^ O-methylhydroxyamine hydrochloride in pyridine at 30°C with continuous shaking. Next, 80 µL N-methyl-N-(trimethylsilyl) trifluoroacetamide was added and the mixture was incubated for additional 30 min at 37°C with continuous shaking. Gas chromatography-mass spectrometry was carried out on a Q Exactive GC Orbitrap GC-tandem mass spectrometer coupled to a Trace 1300 Gas chromatograph (Thermo Fisher Scientific, Waltham, MA, USA). The injector operated in a split mode (inlet temperature 250°C, split ratio 5, purge flow 5.0 mL/min, split flow 6.0 mL/min). Metabolites were separated on a TG-5SILMS GC Column (Thermo Fisher, 30 m × 0.25 mm × 0.25 μm) with helium as a carrier gas at a constant flow of 1.2 mL/min using a 28 min gradient (70°C for 5 min followed by 9°C per min gradient to 320°C and finally 10 min hold time) and ionized using electron ionization mode (electron energy 70 eV, emission current 50 μA, transfer line and ion source temperature 250°C). Mass spectra were recorded by scanning the range of 50 to 750 m/z. Chromatograms were analyzed using TraceFinder 4.1 with Deconvolution Plugin 1.4 (Thermo), Compound Discover (Thermo) and searched against the NIST2014, GC-Orbitrap Metabolomics library, and in-house library. Only metabolites fulfilling identification criteria (score ≥ 75 and ΔRI< 2%) were included in the final list. Quantitative differences were determined by manual peak assignment in Skyline 20.1, using extracted ion chromatograms (2 ppm tolerance).

### Protein extraction and LC-MS analysis

The pellet from the MTBE: MeOH (3:1, v:v) extracted plant sample from metabolite extraction was further used for protein extraction. The pellet was washed with 0.5 ml of MeOH, lyophilized, and resuspended in 0.2-0.5 ml of protein extraction buffer consisting of 8M urea and 100mM ammonium bicarbonate at room temperature until dissolved. Next, aliquots corresponding to 100 μg of protein were reduced, alkylated with iodoacetamide, digested with trypsin (1:100, Promega) and desalted by C18 SPE. Finally, aliquots corresponding to 2.5 μg of peptide were analyzed by nanoflow C18 reverse-phase liquid chromatography using a 15 cm column (Zorbax, Agilent), a Dionex Ultimate 3000 RSLC nano-UPLC system (Thermo) and the Orbitrap Fusion Lumos Tribrid Mass Spectrometer, as described previously (Kameniarová et al., 2022; Saiz-Fernández et al., 2022). The measured spectra were recalibrated and searched against Araport 11 protein database by Proteome Discoverer 2.5, employing Sequest HT and MS Amanda 2.0 with the following parameters: Enzyme-trypsin, max two missed cleavage sites; MS1 tolerance-5 ppm; MS2 tolerance-15 ppm (MS Amanda), 0.1 Da (Sequest, Mascot); Modifications-carbamidomethyl (Cys) and up to three dynamic modifications including Met oxidation, Asn/Gln deamidation, N-terminal acetylation. Only proteins with at least two unique peptides were considered for the quantitative analysis. The resulting data were normalized and analyzed using MetaboAnalyst 5.0 online tool (Xia et al., 2009; Pang et al., 2021). The mass spectrometry proteomics data have been deposited to the ProteomeXchange Consortium *via* the PRIDE (Perez-Riverol et al., 2022) partner repository with the dataset identifier PXD034173

### RNA isolation and qRT-PCR analysis

Total RNA was isolated by using RNeasy Plant Mini Kit (Qiagen). The RNA quality and quantity were assessed by NanoDrop 2000 spectrophotometer (Thermo Fisher Scientific, USA). cDNA was prepared using 1.0 µg of total RNA using RevertAid Reverse Transcriptase (Thermo Fisher Scientific, USA). qRT-PCR analysis was performed with 1:10 diluted cDNA samples using Luna^®^ Universal qPCR Master Mix (New England Biolabs) on LightCycler 480 II (Roche, Germany) Real-Time PCR System. The primers used were designed using QuantPrime qPCR primer design tool (Arvidsson et al., 2008). The primers are listed in **Supplementary Table S6**. The relative quantification of mRNA level was calculated using the ΔΔCT method (Livak and Schmittgen, 2001).

### Quantification of glutathione and ascorbate

The levels of glutathione and ascorbate were measured by the method described by (Queval and Noctor, 2007). Briefly, approximately 100 mg tissue derived from the whole seedlings grown under standard growth conditions was homogenized in 0.2 M HCl. After centrifugation, the pH of the resulting supernatant was adjusted to 5.0 by sodium phosphate buffer and 0.2 M NaOH. The total and oxidized glutathione and ascorbate were then quantified by the plate-reader assay.

## Data availability statement

The datasets presented in this study are publicly available at the ProteomeXchange repository with the accession number: PXD034173, http://proteomecentral.proteomexchange.org/cgi/GetDataset?ID=PXD034173.

## Author contributions

SJ, BB, and AL conceived the study. SJ, BB, and PK designed experiments. SJ and HKB performed thermotolerance assays. SJ, PK, and MČ performed proteomics studies and analysed data. SJ, PK, and MB performed metabolomics study. SJ performed all other experiments. SJ wrote the paper and BB and MČ reviewed the paper. BB and SJ revised the manuscript. BB acquired funding. All authors have read and agreed to the published version of the manuscript.

## Funding

This work was supported by Ministry of Education, Youth and Sports of the Czech Republic (European Regional Development Fund-Project “Centre for Experimental Plant Biology” (Grant no. CZ.02.1.01/0.0/0.0/16_019/0000738) and the Internal Grant Schemes of Mendel University in Brno. Reg. no. CZ.02.2.69/0.0/0.0/19_073/0016670, funded by the ESF.

## Conflict of interest

The authors declare that the research was conducted in the absence of any commercial or financial relationships that could be construed as a potential conflict of interest.

## Publisher’s note

All claims expressed in this article are solely those of the authors and do not necessarily represent those of their affiliated organizations, or those of the publisher, the editors and the reviewers. Any product that may be evaluated in this article, or claim that may be made by its manufacturer, is not guaranteed or endorsed by the publisher.
