# Supplementary figures and images for: Type-A response regulators negatively mediate heat stress response by altering redox homeostasis in Arabidopsis
Source: Front Plant Sci. 2022 Sep 23;13:968139. doi: 10.3389/fpls.2022.968139 (PMC9539118; doi:10.3389/fpls.2022.968139)

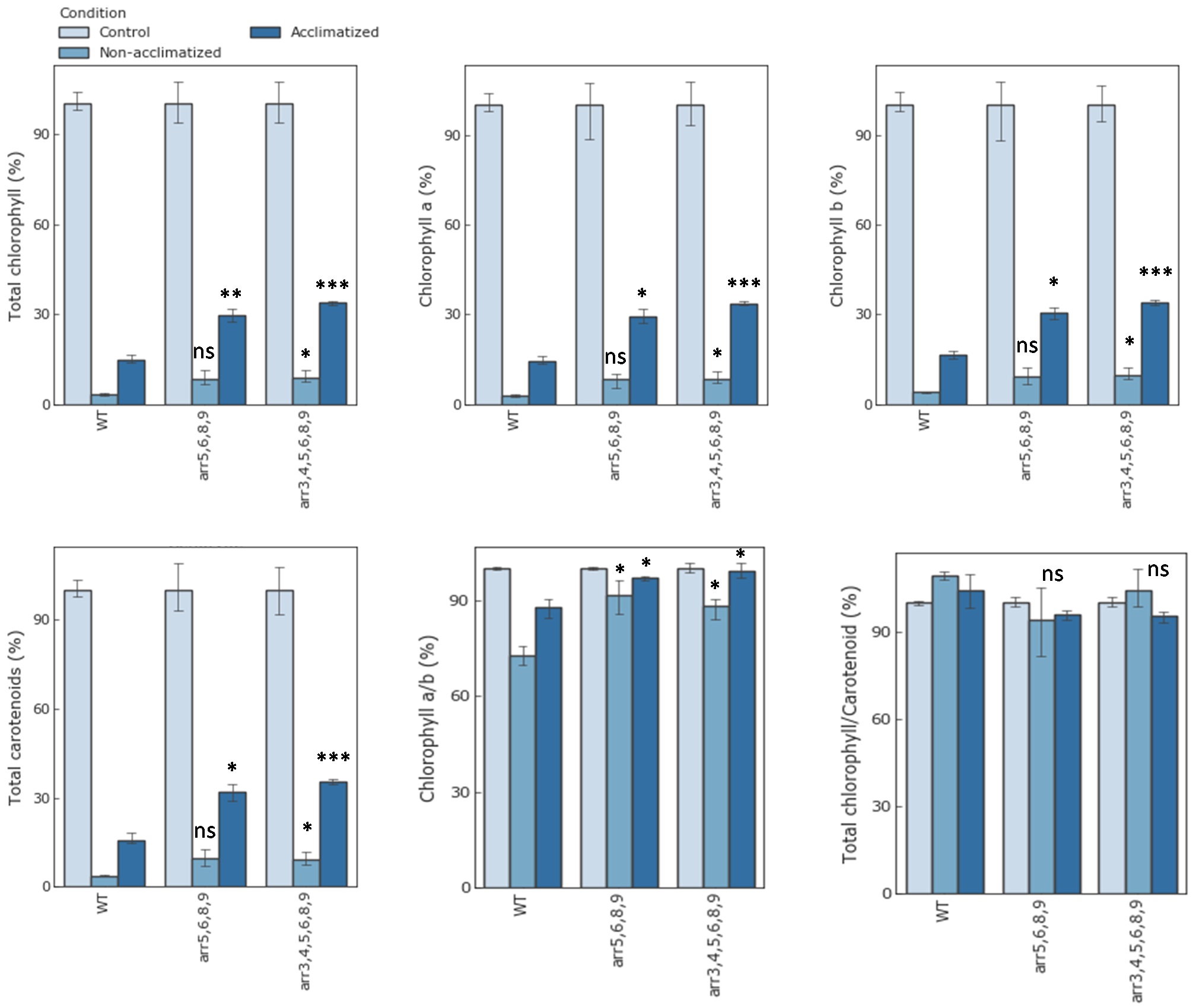

Supplement: Supplementary file 1 [file Image_1.jpeg]

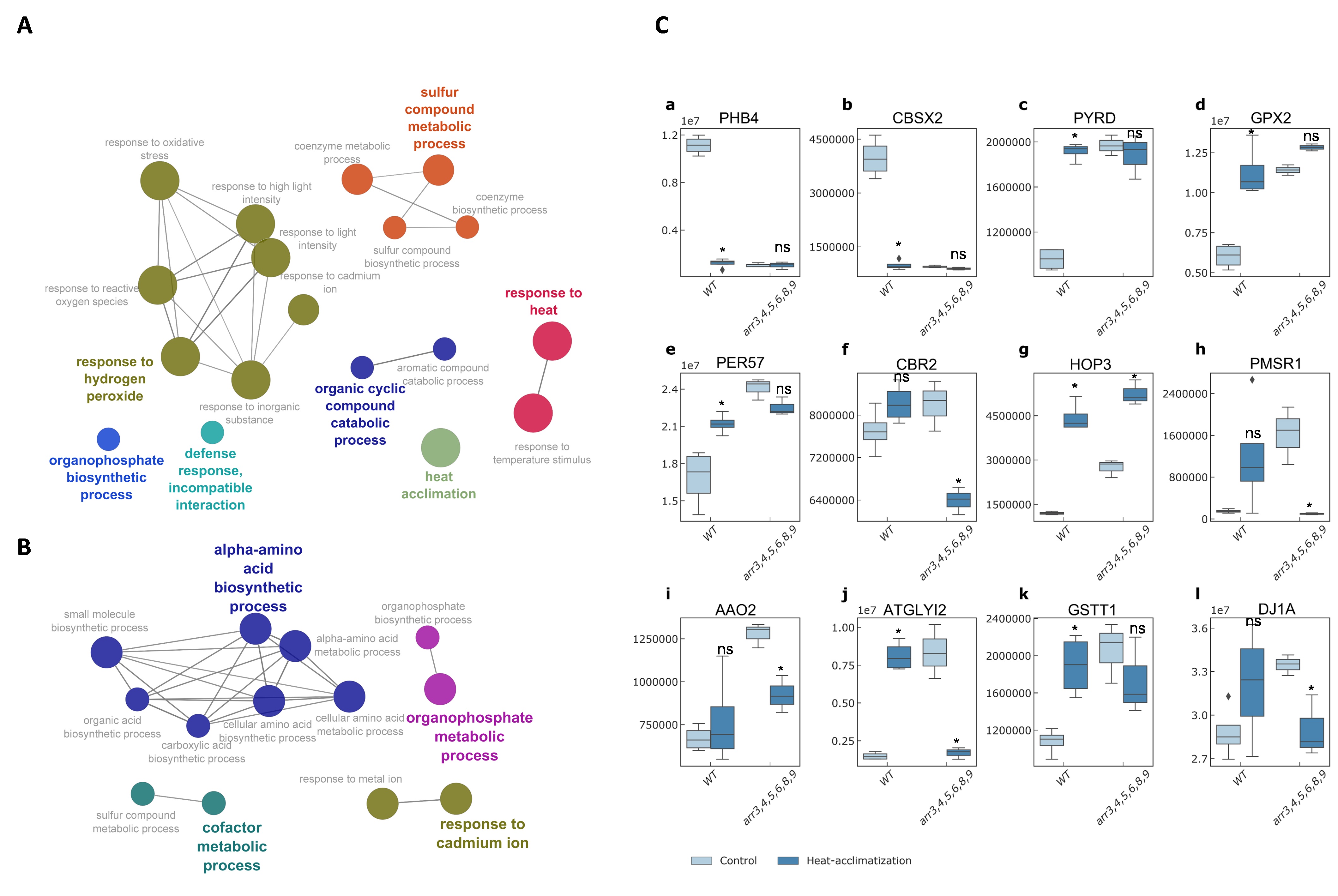

Supplement: Supplementary file 2 [file Image_2.jpeg]

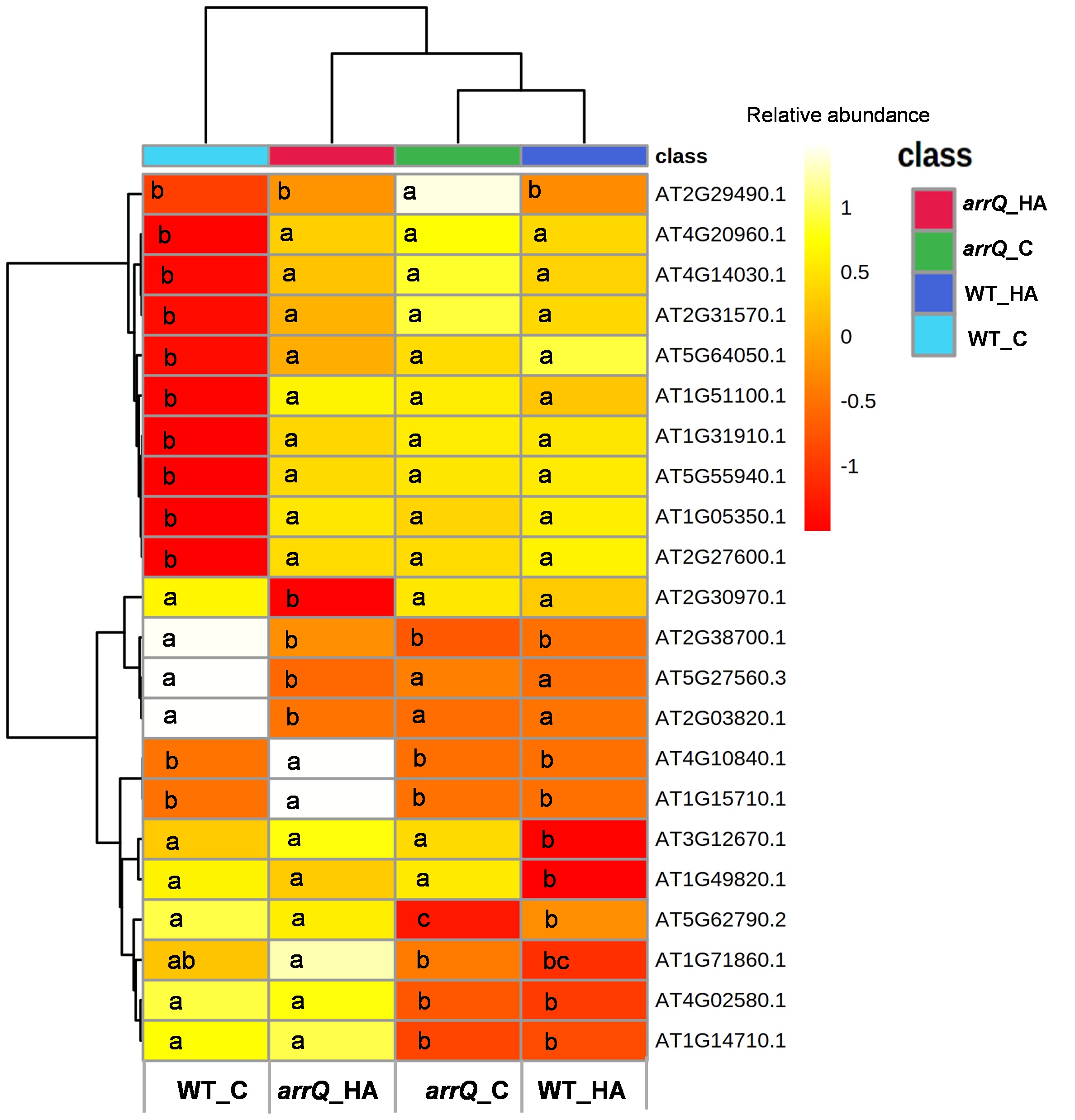

Supplement: Supplementary file 3 [file Image_3.jpeg]

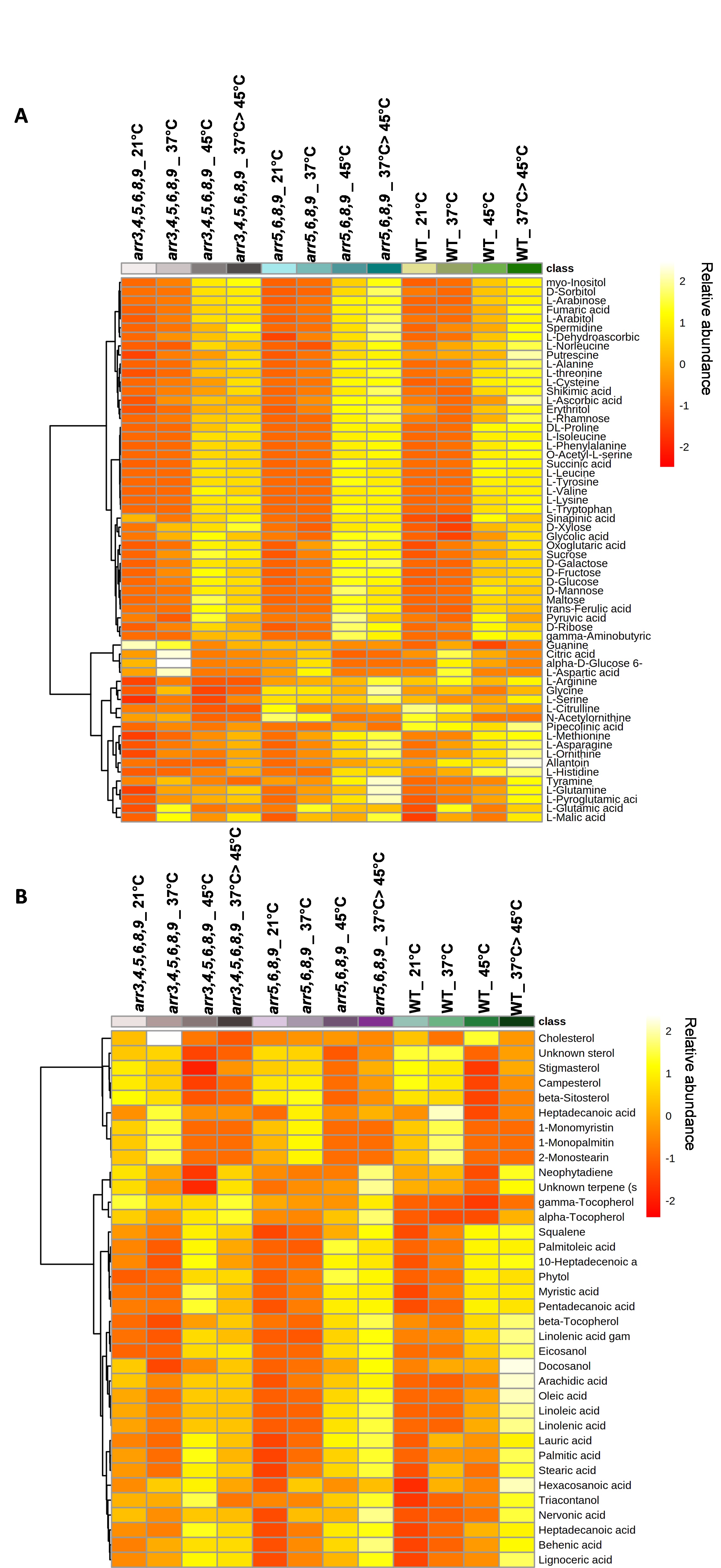

Supplement: Supplementary file 4 [file Image_4.jpeg]

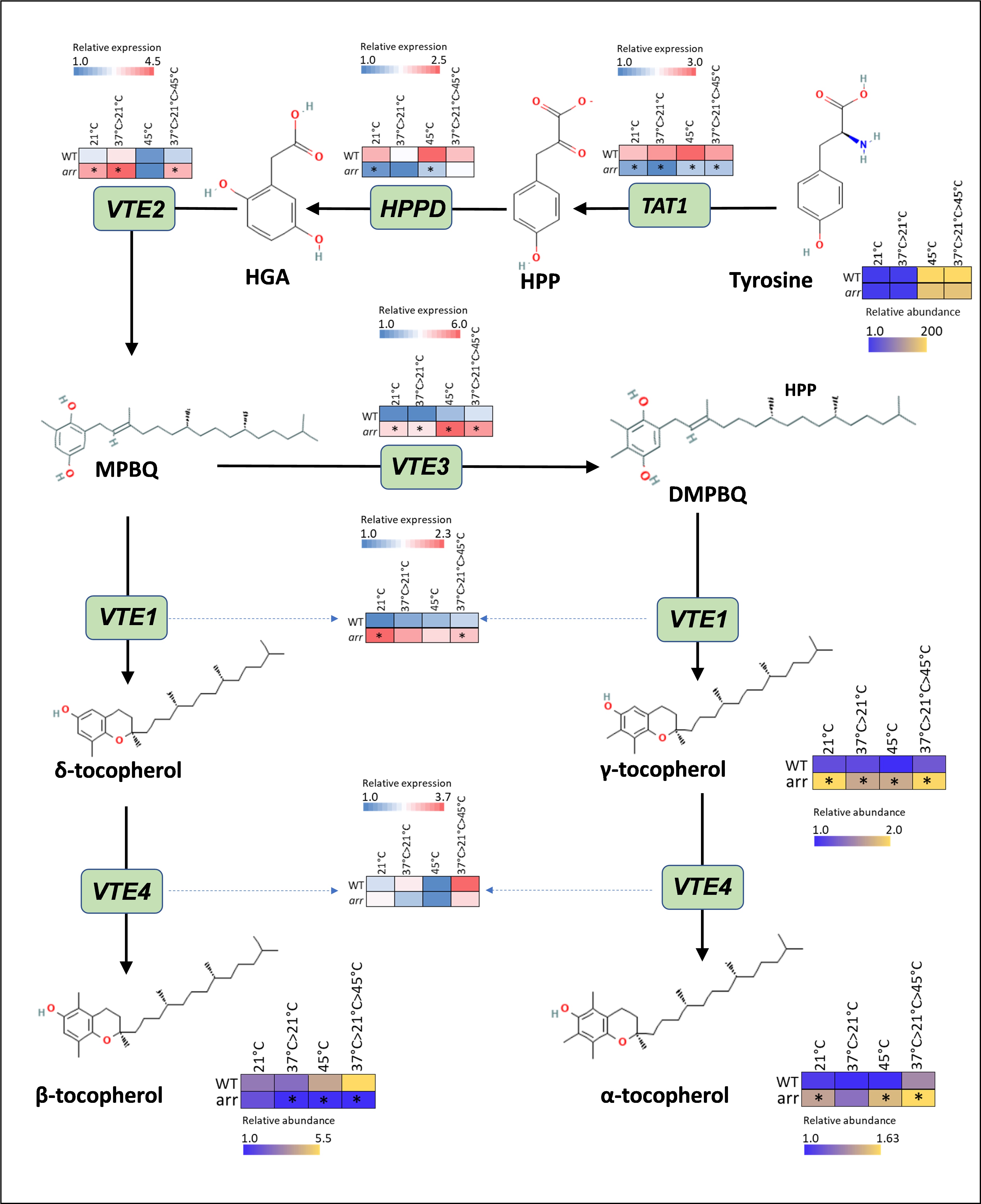

Supplement: Supplementary file 5 [file Image_5.jpeg]

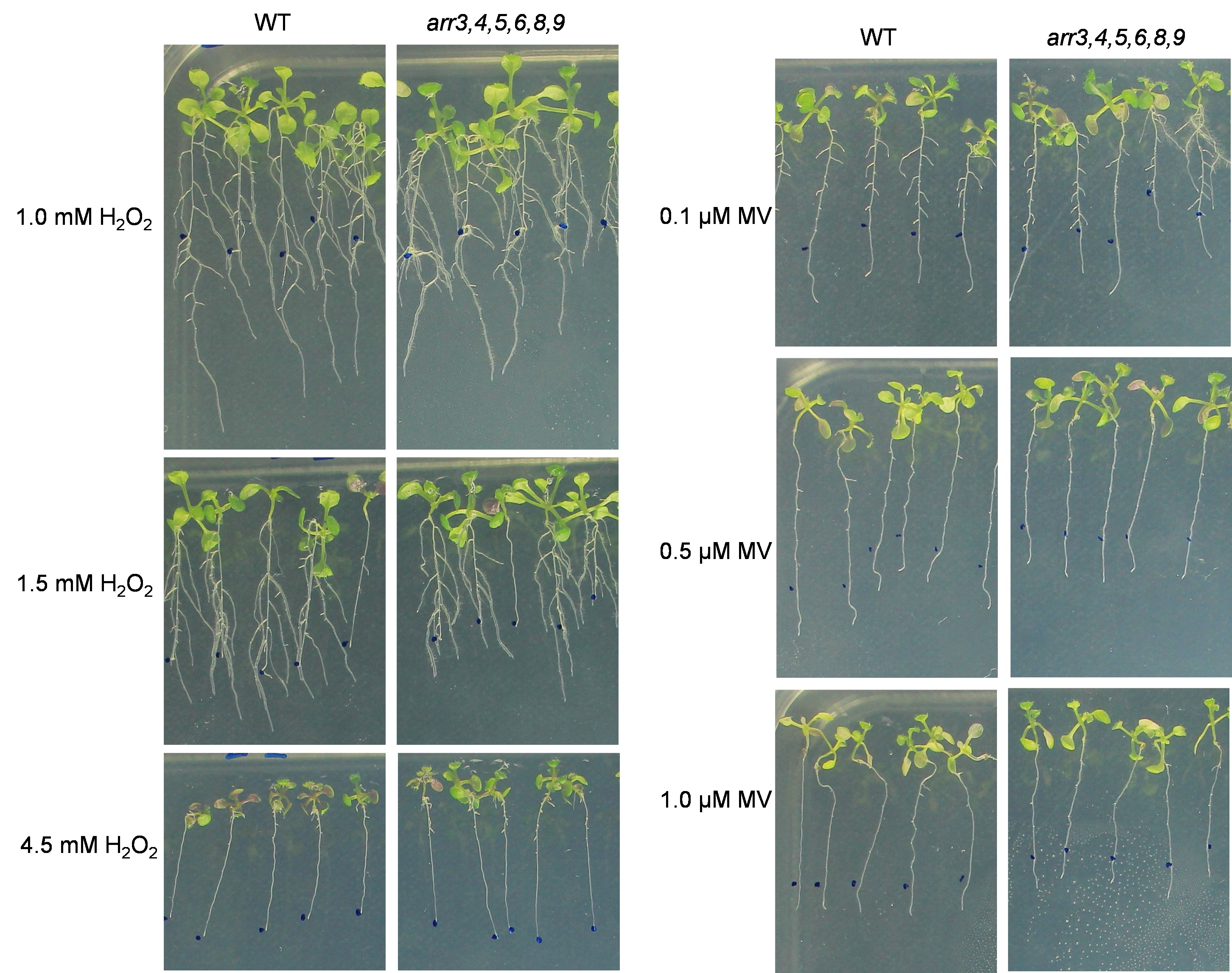

Supplement: Supplementary file 6 [file Image_6.jpeg]
