# Supplementary material for: Type-A response regulators negatively mediate heat stress response by altering redox homeostasis in Arabidopsis
Source: Front Plant Sci. 2022 Sep 23;13:968139. doi: 10.3389/fpls.2022.968139 (PMC9539118; doi:10.3389/fpls.2022.968139)
Supplement: Supplementary file 8 [file Table_2.docx]

Supplementary Material

1. **Supplementary Figures**

**Figure S1. Differential accumulation of photosynthetic pigments in the wild-type and *arr* mutants under control and heat stress conditions. Related to Figure 2.**

Histograms representing total chlorophyll content, chlorophyll a, chlorophyll b, total carotenoids, chlorophyll a and b ratio, and the ratio of total chlorophyll and carotenoid. The contents are normalized with respect to the control group of each genotype to show the relative changes. The statistical significance is shown between the WT and *arr* mutants within the same treatment group. Error bars represent ±SE (two-way ANOVA, *P-value ≤ 0.05, Bonferroni post-hoc test, n=3, 13-15 plants were taken for each biological replicate.

**Figure S2. Overview of the proteomics dynamics following a heat-acclimatization phase. Related to Figure 4.**

(A) Enriched functional clusters of the DAPs in WT seedlings after heat-acclimatization. (B) Enriched functional clusters of the proteins showing interaction in two-way ANOVA (genotype x treatment) at P-value ≤ 0.05. The network visualization is done in Cytoscape v3.9.0 plugin Clugo. The nodes represent the GO terms and the size of the node represents term enrichment significance. The leading group name is based on the highest significance and only the pathways with P-value ≤ 0.05 are shown. (C) Graphical representation of the selected proteins showing interaction effect in two-way ANOVA (genotype x treatment) [Control vs. acclimatized, genotypes *arr3,4,5,6,8,9* vs WT]. The statistically significant differences between the heat-acclimatized and control samples of each genotype are indicated by an asterisk. Treatments were performed in at least 3 biological replicates. P-value ≤ 0.05, ns not significant.

**Figure S3. Proteomics changes triggered by heat-acclimatization in WT and *arr5,6,8,9* mutant. Related to Figure 4.**

Heat map view of DAPs upon heat-acclimatization/mild heat treatment. Only the proteins that show statistical interaction effect in two-way ANOVA (genotype x treatment) are shown in the heat map and the letters on the heat map indicate the statistical difference in Turkey’s test. The protein identities are listed on the right. The heatmap scale indicates relative abundance. Treatments were performed in at least 3 biological replicates. The heatmap visualization and hierarchical clustering are carried out using MetaboAnalyst 5.0. ***arrQ*_C**: *arr5,6,8,9*_Control; ***arrQ*_HA**: *arr5,6,8,9*_Heat-acclimatized; **WT_C**: WT_Control; **WT_HA**: WT_Heat-acclimatized

**Figure S4. Overview of the metabolomic response to high temperature. Related to Figure 5.**

Heat map visualization of (A) polar metabolites. (B) non-polar metabolites in the WT and *arr* mutants. (Treatments: 21°C - untreated control grown at 21°C; 37°C -acclimatization at 37°C for 1 h followed by 2 h of recovery at 21°C; 45°C - heat stress at 45°C for 2.5 h; 37°C>45°C - acclimatization at 37°C for 1 h followed by 2 h of recovery at 21°C and heat stress at 45°C for 2.5 h).

**Figure S5. Tocopherol biosynthetic pathway genes are differentially expressed in the type-A *arr* mutant. Related to Figure 5.**

Tocopherol biosynthetic pathway in Arabidopsis and its differential regulation in the type-A *arr* mutant. The relative expression levels of *TAT1*, *HPPD*, *VTE1*, *VTE2*, *VTE3*, and *VTE4* are shown by the heat maps above the respective genes. The treatment conditions are shown on the top of the heat maps (21°C - untreated control grown at 21°C; 37°C>21°C -acclimatization at 37°C for 1 h followed by 2 h of recovery at 21°C; 45°C - heat stress at 45°C for 2.5 h; 37°C>21°C >45°C - acclimatization at 37°C for 1 h followed by 2 h of recovery at 21°C and heat stress at 45°C for 2.5 h). The *arr3,4,5,6,8,9* mutant is shown as *arr*. The asterisk shows the statistically significant difference between the WT and *arr* in one-way ANOVA. P-value ≤ 0.05. The relative abundance of the metabolites tyrosine (L-tyrosine), α-, β-, and γ-tocopherols are shown in the heat map.

**Figure S6. Oxidative stress response in the absence of functional type-A ARRs. Related to Figure 6.**

7 DAS seedlings grown on 0.5 X MS medium were transferred to the medium containing methyl viologen (MV) or H_2_O_2_ and allowed to grow in the same plates for 3 weeks. The images shown are captured after 1 week of the transfer to the media containing MV and H_2_O_2_. The treatments were performed in 4 biological replicates.

**2. Supplementary Tables**

**Supplementary Table S1** DAPs in *arr3,4,5,6,8,9* compared to WT

**Supplementary Table S2** DAPs in *arr5,6,8,9* compared to WT

**Supplementary Table S3** DAPs in WT after heat-acclimatization

**Supplementary Table S4** Proteins showing interaction in two-way ANOVA (genotype x treatment) [Control vs. acclimatized, genotypes *arr3,4,5,6,8,9* vs WT]

**Supplementary Table S5** Redox-related DAPs in *arr5,6,8,9* and *arr3,4,5,6,8,9* compared to WT

**Supplementary Table S6** List of primers used for qRT-PCR
